# Participants’ perspectives of “NeuroSask: Active and Connect”—a virtual chronic disease management program for individuals with a neurological condition

**DOI:** 10.3389/fneur.2024.1332859

**Published:** 2024-01-24

**Authors:** Stephen E. Patrick, Katherine B. Knox, Charity Evans, Michael Levin, Gary Linassi, Ilia Poliakov, Alex Rajput, Sarah J. Donkers

**Affiliations:** ^1^Department of Physical Medicine and Rehabilitation, College of Medicine, University of Saskatchewan, Saskatoon, SK, Canada; ^2^College of Pharmacy and Nutrition, University of Saskatchewan, Saskatoon, SK, Canada; ^3^Anatomy and Cell Biology, College of Medicine, University of Saskatchewan, Saskatoon, SK, Canada; ^4^Office of the Saskatchewan Multiple Sclerosis Research Chair and Division of Neurology, Department of Medicine, College of Medicine, University of Saskatchewan, Saskatoon, SK, Canada; ^5^Multiple Sclerosis Clinic, Division of Neurology, Department of Medicine, College of Medicine, Multiple Sclerosis Clinic, University of Saskatchewan, Saskatoon, SK, Canada; ^6^Movement Disorders Program, Division of Neurology, Department of Medicine, College of Medicine, University of Saskatchewan, Saskatoon, SK, Canada; ^7^School of Rehabilitation Science, College of Medicine, University of Saskatchewan, Saskatoon, SK, Canada

**Keywords:** chronic disease, exercise, neurological rehabilitation, self-management, telerehabilitation

## Abstract

**Introduction:**

Neurological conditions account from more than half of Canadians requiring chronic care. Both physical activity and the development of a self-management skillset are critical components supporting individuals with chronic health conditions. “NeuroSask: Active and Connected” is a virtual chronic disease management program offering twice weekly neuro-physiotherapist directed “active” exercise sessions, followed by weekly knowledge-exchange “connect” sessions with invited guest experts. NeuroSask was launched April 2020 in response to the restricted services and supports for people with neurological conditions. The program aimed to provide seated physical activity, social interaction, and access to expertise in neurological conditions and neurorehabilitation. A program evaluation of NeuroSask was conducted to gain participants’ perspectives.

**Methods:**

All participants registered for the NeuroSask program were invited to complete optional online surveys (SurveyMonkey) circulated by email at 3 occasions post-program launch: 10 weeks, 1 year, and 2 years. Participants could complete any one or all of the surveys, at their discretion. The number of potential respondents changed dependent on the total number of participants registered for NeuroSask at the time the survey was circulated. Questions were co-designed by multi-stakeholder team members. Descriptive statistics were used for closed-ended questions and a reflexive thematic analysis was completed with coding conducted in NVivo 12 Plus for open-ended text.

**Results:**

Response rates (participants/registrants) were as follows: 10-week survey 260/793, one year survey 326/1224, and 2-year survey 434/1989. 90% of participants reported being in either the age categories of 40–59 years or above 60 years. 75% of both survey respondents and program registrants were female. 70% of both survey respondents and program registrants reported a diagnosis of multiple sclerosis and 30% reported other neurological conditions. Survey respondents were from all ten Canadian provinces, with 45% reporting living outside of large cities. Respondents reported preferring online vs. in person format for this type of programming. Three main themes, and eight corresponding subthemes were identified highlighting the perceived impact and key components of the NeuroSask program: Theme 1 “together in a positive and encouraging environment” (subthemes 1a: connection, 1b: empowerment); Theme 2 “access to enthusiastic qualified leaders from home” (subthemes 2a: leader characteristics, 2b: accessibility, 2c: program logistics); Theme 3 “being able to enjoy everyday life” (subthemes 3a: symptom benefits and beyond, 3b: carry-over, 3c: keep going, please do not cancel).

**Conclusion:**

NeuroSask is an example of an accessible and meaningful virtual approach to providing ongoing support for some individuals with neurological conditions. It was perceived as beneficial for fostering community and connection in a positive environment with perceived benefits extending beyond symptom management to participant reported improvements in function, daily life, and disease experience.

## 1 Introduction

Neurological conditions are among the leading causes of disability resulting in long-term challenges with activities of daily living and participation in meaningful life tasks (1–4). Mood disorders and secondary medical complications are also highly prevalent among this population (4). Neurological conditions account for more than half of Canadians requiring chronic care, where ongoing symptom management support is required (5). Development of a self-management skill-set is a critical component supporting optimal health outcomes for people with neurological conditions. Research shows that an individual’s everyday management of their condition and its consequences has a profound impact on their current and future health and well-being (4–8). Commonly reported challenges for people with neurological conditions include access to both health care specialists with experience in the unique care needs of individuals with neurological conditions and the supports for effective self-management (2, 9).

Gaining self-management skills (e.g., core knowledge, self-awareness, self-monitoring, goal-setting, action planning etc.) is a critical component influencing health behavioural change (4). Knowledge of ways to best support individuals with neurological conditions in developing self-management skills is crucial to delivering high quality chronic disease management services and an important step in advocating for such supports to be integrated into routine care (10, 11). Novel approaches for delivering self-management support for lifestyle and symptom-management behaviours need to be explored (4, 7–10, 12). Further, health care systems are increasingly faced with resource challenges to deliver services and supports for people living with neurological conditions, with the pandemic imposing even greater system challenges (13–15). In response to these gaps, the virtual NeuroSask Active and Connected Program was developed in Saskatchewan, Canada, in April of 2020.

NeuroSask was collaboratively designed as a community partnership program specifically for people with neurological conditions. The program aimed to provide support for self-management, two-way knowledge exchange (between neurological and rehabilitation health care experts and those with lived experience), and to create a venue to nourish social interaction and regular physical activity as key components of ongoing symptom management. NeuroSask was originally intended to run a total of ten weeks, during the time period many of the health care system and community services for people living with neurological conditions were closed due to the pandemic onset. However, due to strong participant advocacy for the NeuroSask program to continue, MS Canada provided extended program funding support. The NeuroSask Active and Connected program continues to run. The aim of this study was to evaluate the “NeuroSask” program from the participants’ perspectives.

## 2 Materials and methods

As part of a program evaluation of NeuroSask, online feedback surveys were used to capture participant perspectives. A survey (see Appendix 1) was circulated on 3 occasions: 10 weeks, 1-year, and 2-years post program launch. Participants could complete any one or all of the surveys, at their discretion. Voluntary surveys were distributed by email with a link to SurveyMonkey.^1^ At each survey occasion, one email invitation was sent to all the participants registered with the NeuroSask program. The number of potential respondents changed dependent on the total number of participants registered for NeuroSask at the time the survey was circulated. The survey questions were collaboratively designed with the multi-stakeholder team through discussion at our regular team meetings. Questions were designed based on the type of information the different stakeholders felt necessary to inform ongoing programming. The survey was completed by patient advisors and further discussed by team before circulating. The initial survey was circulated at 10 weeks after the launch of the program, during the COVID-19 pandemic while many rehabilitation services were still on lockdown. Subsequently, this survey was concise and designed to inform the team, including community partners, of participant interest as well as gauge participation rates across demographics. The second and third survey time points (one-and two-years post launch) included more questions about perceived benefit and program logistics to inform future program development. In the third survey, we inquired specifically about frequency of participation. Since the NeuroSask program was launched initially as a 10-week pilot, the continuation of the program was unknown each time the survey was circulated.

All surveys included a mixture of closed-ended (e.g., multiple choice, select all that apply click-box questions, sliding scale answers) and open text box answers. A quantitative descriptive approach was used to report the results of closed-ended questions, and a reflexive thematic analysis approach was applied for the open text field questions (16, 17). The University of Saskatchewan Biomedical Research Ethics Board was consulted on the implementation of the NeuroSask: Active and Connected community program and the methods to collect participant perspectives about the program. A waiver of ethics was approved by the University of Saskatchewan Research Ethics Board as the work was deemed to be about program evaluation and quality improvement. The survey also asked for participant consent to anonymously share the results. All responses were anonymously collected through the online survey platform, and remained anonymous through the review, analysis, and writing process.

### 2.1 Participants

All participants who registered for the NeuroSask program online (regardless of their attendance at the NeuroSask Zoom sessions) were invited to voluntarily provide feedback through the surveys.

The NeuroSask program was designed to target individuals with moderate mobility impairments from a neurological condition, offering an open, unrestricted online self-registration process. The program has a rolling intake and runs on a drop-in basis. The program was advertised by media releases about the program, by the non-profit collaborating partners (e.g., Multiple Sclerosis Society), referral from health care providers or through a link on the website of the College of Medicine, University of Saskatchewan. Upon registration (completed via an online portal), participants are asked to self-report their age range, sex, Canadian province of primary residence (or other country of residence), and report if their place of residence is rural or non-rural. In order to protect confidentiality, place of residence options are restricted to a drop-down list with the names of the Canadian provinces, or “other” if the participant does not reside in Canada. “Prefer not to say” is an option for all questions. Participants are also asked to report which neurological condition they are living with (e.g., multiple sclerosis, spinal cord injury, Parkinson’s disease, stroke etc.). To complete registration, participants must agree to statements acknowledging that the program is a general educational program, that they will not be provided with individualized medical care, and that they are participating at their own risk. Finally, participants provide an email to receive the on-going reminder and unique Zoom link for each class. Participants can unregister at any time on their own accord.

### 2.2 Intervention, the “NeuroSask: Active and Connected” program

The NeuroSask Active and Connected program was initially developed in response to a call from the Saskatchewan Health Research Foundation (a provincial funding agency) for “rapid response” pandemic projects. The program was developed as a partnership amongst researchers, health care providers, decision-makers, community-based organizations, and people with lived experience. Members of the leadership team met virtually to co-design and provide input on the NeuroSask program. Our team consisted of researchers from the University of Saskatchewan, physicians and physical therapists specializing in multiple sclerosis (MS), Parkinson’s disease and spinal cord injury care, a manager working within the health authority, people living with a neurological condition, and key representatives from MS Canada, Parkinson’s Canada, and Spinal Cord Injury Saskatchewan. The co-design process involved real-time group discussion (virtually) with some in session anonymous polling done for decision making (e.g., time, length, and frequency of sessions for the program). Agendas and any corresponding material were pre-circulated so that all members of the team had time to reflect on proposed topics of discussion and content, with the opportunity to add to the agenda. Once the components of the program were decided, details for the “active” and “connected” portions were discussed. For the active portion, a sample class was designed and delivered for the team (including patient advisors) for feedback, and to test out the challenge level, speed, flow, and set-up (e.g., sound, camera placement). For the connect session, initial discussions included brainstorming among the leadership team on guest experts and topics that would be relevant to people living with neurological conditions during the pandemic. Further programming for the connect sessions was largely based on participant feedback and requests.

The NeuroSask Active and Connected program offers two integrated sessions: “active” sessions involving thirty minutes of seated movement led by an experienced neuro-physiotherapist, followed by a thirty minute “connect” session. “Active” sessions are designed to enhance body awareness, symptom management and functional mobility as well as provide education about the importance of physical activity and ways to be active around the home. Movements focus on alignment, seated postural control (e.g., reaching and weight shifts), limb and trunk range of motion, with some resistance training and bouts of cardio sprinkled throughout. The active sessions have a template of components (e.g., warm-up options, cardio options, resistance exercises for upper and lower limbs, trunk movements etc.) that vary session to session. Options for different levels of challenge or movement abilities within each exercise are given (e.g., level 1, 2, 3), and increases in movement challenges or range of motion are layered in by instructor (i.e., after a couple repetitions of the base movement pattern). The seated nature allows us to be inclusive of participant physical ability level and to minimize the risk of falls. In addition to the person leading the active portion, another team member is present to help monitor and run the platform (e.g., monitor chat and the other interactive Zoom functions participants might use like reactions or raise hand, scan participant list and cameras for any questions or safety concerns, ensure sound quality of instructor, and monitor the email in case anyone reports concerns or challenges with accessing the session). “Connect” sessions are designed as educational and self-management support opportunities with the intention to cover topics on neurology, neurorehabilitation, and health and wellness (18, 19) (e.g., resiliency, mindfulness, creativity, art-based therapy, etc.). Connect sessions include a mixture of discussions, questions and answers, reviews of the evidence, guided-learning activities (e.g., goal setting, action planning, completing a symptom monitoring diary), experiential learning (e.g., guided mindfulness), and “tips and tricks” on topics facilitated by invited leading experts in their field. Selected volunteer connect guests are content experts who hold affiliations with non-profit, community, health care or academic institutions. The connect sessions are moderated by the NeuroSask project leads (SJD and/or KK). The moderators are clinician researchers knowledgeable in self-management support and health related behavior change (SJD), and with expertise in neurorehabilitation (SJD, KK).

The program runs twice a week on Tuesdays and Thursdays at 1400 h, Central Standard Time. Registered participants receive an email every Monday and Wednesday evening with a reminder and unique Zoom link for the following Tuesday or Thursday session. The Zoom meeting platform allows participants to raise their hand to be un-muted by the moderator, and participants are invited at any time to share comments, experiences, and questions in the chat. On the Zoom meeting platform, participants can choose to use their real or an alias name, and whether to have their cameras on. At the end of each connect session, the moderator allows participants to be able to un-mute themselves and they are invited to stay on the Zoom meeting platform for informal social interaction. Participants are encouraged to make suggestions/requests for the connect topics and/or future guests. Moderators make every effort to fulfill these requests with a qualified and experienced guest (e.g., specialized health care provider or researcher in topic area). All sessions are live on the virtual platform and not recorded to encourage real-time engagement. Participation in any session is voluntary. Anyone interested in learning more about the NeuroSask program can contact corresponding author.

### 2.3 Analysis

Survey response rates for each survey, participant demographics and all closed-ended questions (e.g., sliding scale) survey responses were analyzed using descriptive statistics. Survey response rates for each of the three surveys were calculated according to the number of participants who completed the survey and the total number of participants registered for NeuroSask at the time the survey was circulated. Percentages were calculated from the returned surveys to describe the age ranges, gender, primary provincial residence of the participant, whether they identified as living in a rural versus non-rural community and if they identified as a person with a neurological condition, or other. Mean and standard deviation scores were calculated for sliding scale survey responses related to participant perspectives on aspects of the program content and delivery.

For the open text survey questions, qualitative analysis was conducted using reflexive thematic analysis approach described by Braun and Clarke (16, 17). The process of analysis included familiarization with the dataset by reading through the open-text results multiple times. Preliminary coding was developed independently by each analyzing researcher (SP, SJD, KK). Keywords were co-developed based on discussion and keywords were grouped to develop preliminary themes. Data were reviewed several times in the process of refining, defining, and labelling final themes. Finally, results were shared with a patient advisory group of individuals living with neurological conditions to review and inform wording. All responses remained anonymous through the data collection, analysis, and writing process. Although outlined sequentially, each analysis phase builds on the previous but uses an iterative approach with movement back and forth between the phases. Authors SJD, KK and SP conducted the thematic analysis. Author SP was not involved with any of the NeuroSask Program design or implementation process. Authors SJD and KK are Co-PI investigators for the development of the NeuroSask program and continue to co-lead the program.

All three authors reviewed the raw data, coded separately, and met at multiple stages throughout the process to compare, review, and refine themes. Surveys yielded a large volume of open text across all three time-points. Preliminary analysis indicated that the codes were highly consistent across the three survey time points. The consistency of the data informed the decision to compile all the data for the qualitative analysis.

## 3 Results

### 3.1 Quantitative results

**Figure 1:**
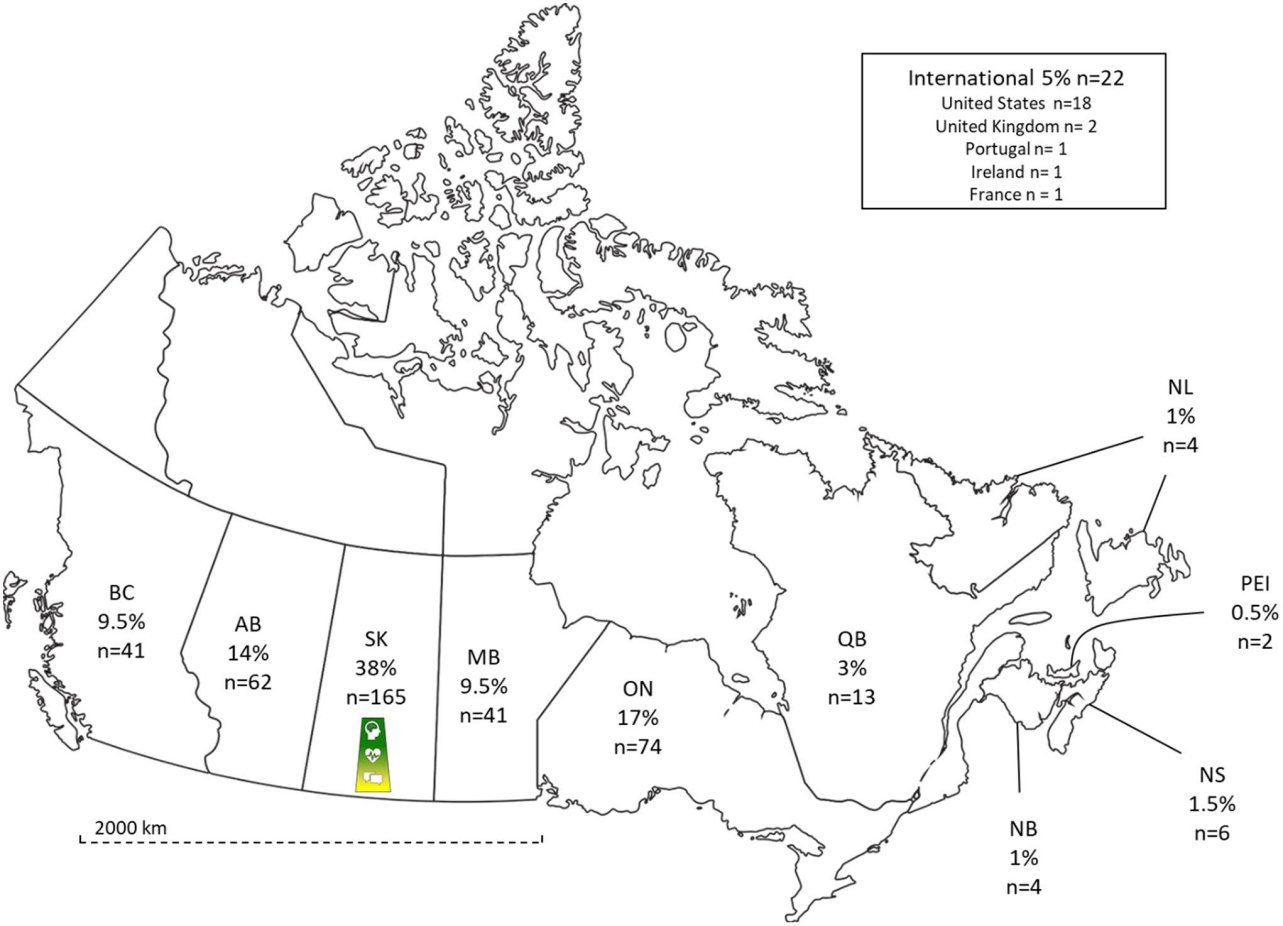
Respondent geographical distribution. Geographical distribution of survey respondents at two years. NeuroSask logo represents site of initiation of NeuroSask (BC, British Columbia; AB, Alberta; SK, Saskatchewan; MB, Manitoba; ON, Ontario; QB, Quebec; NB, New Brunswick; NS, Nova Scotia; PEI, Prince Edward Island; NL, Newfoundland and Labrador).

Survey response rates and demographics are displayed in Table 1. Respondent demographics showed similar proportions to the registrants in the NeuroSask program for each survey time point, e.g., among both the registrants and respondents, 70% reported living with MS. The majority of the remaining 30% of respondents reported living with Parkinson’s disease, spinal cord injury, stroke, cerebral palsy, or cerebellar ataxia. Other demographic data varied slightly across the three survey time points (Table 1). For the two-year survey, respondents included people from all Canadian provinces, as well as some international locations (Figure 1).

**Table 1:** Survey demographics.

	Survey 1:10 weeks post-launch	Survey 2:1 year post-launch	Survey 3:2 years post-launch
Number registered in the NeuroSask program at time of survey (*n*)	793	1,224	1989
Number of survey respondents (*n*)	260	326	434
Sex	Female: 84%	Female: 70%	Female: 80%
Age range (percentage)	<20 years: 2%	<20 years: 2%	<20 years: 0%
	20–39 years: 8%	20–39 years: 5%	20–39 years: 10%
	40–59 years: 45%	40–59 years: 47%	40–59 years: 30%
	>60 years: 45%	>60 years: 46%	>60 years: 60%
Location: (rural, small or large city)	Rural: 20%	Rural: 20%	Rural: 17%
	Small City: 25%	Small City: 25%	Small City: 29%
	Large City: 55%	Large City: 55%	Large City: 53%

**Figure 2:**
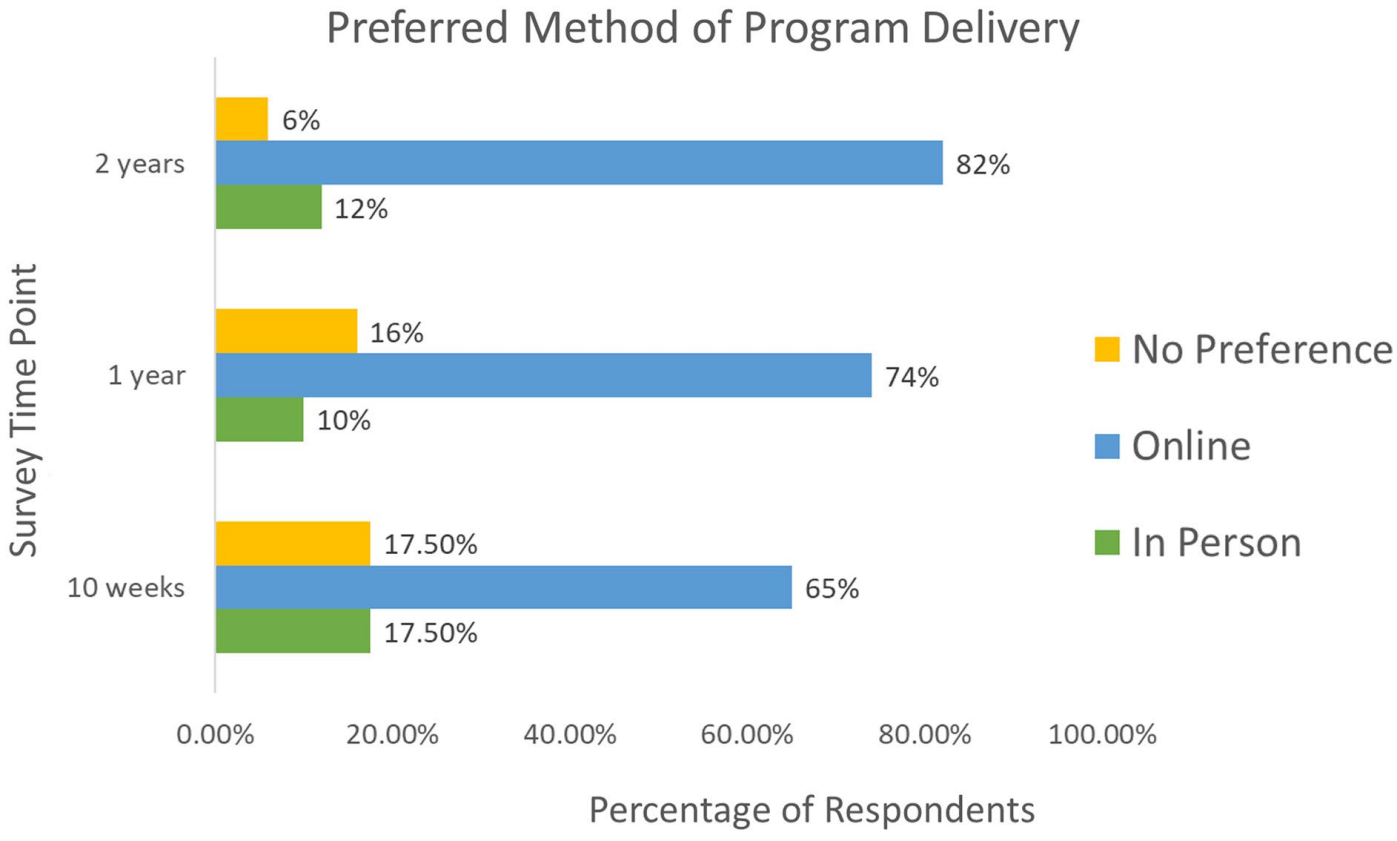
Respondent preferred method of delivery for the NeuroSask Active and Connected program across three time points.

**Figure 3:**
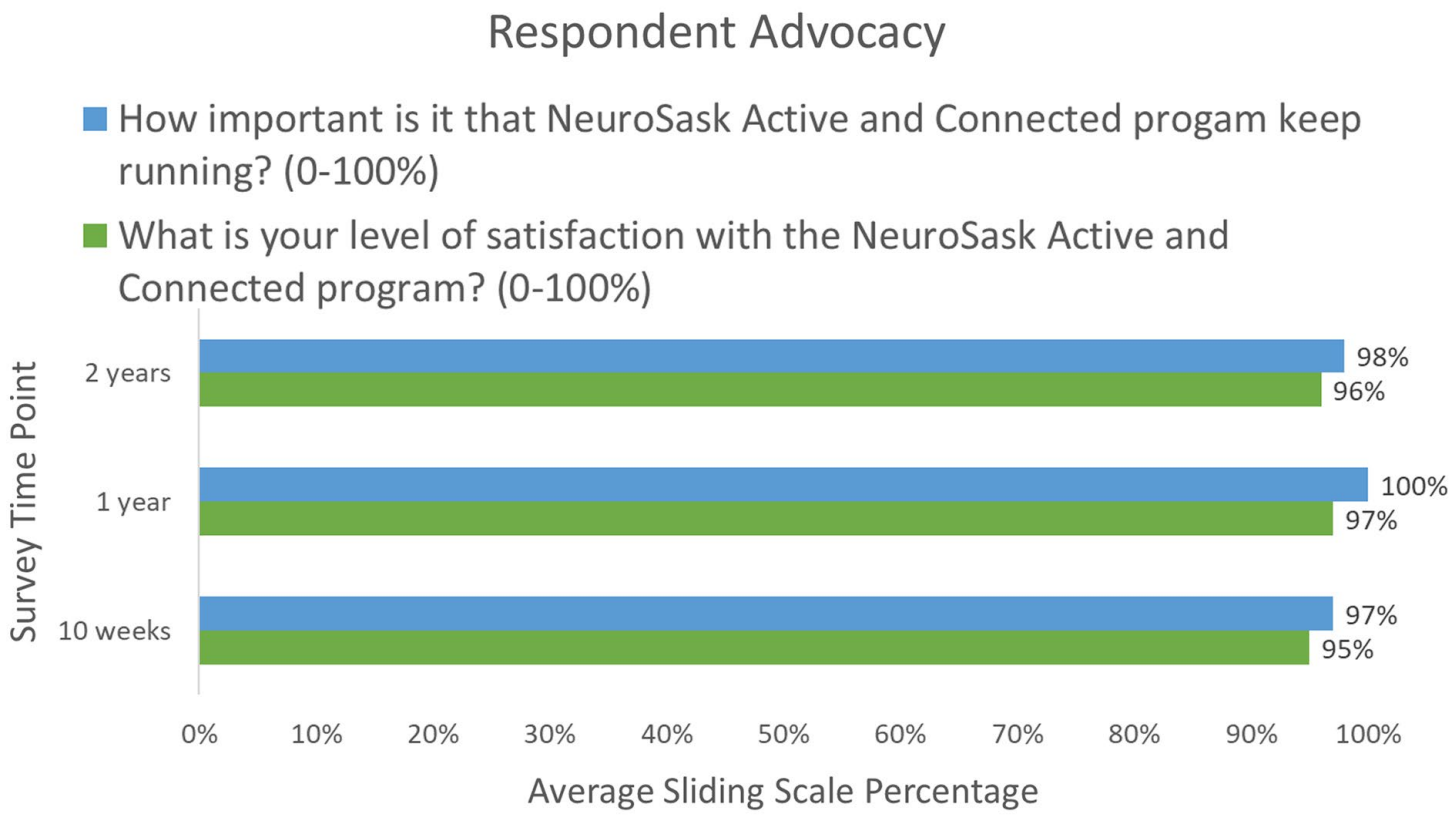
Respondent survey results for closed-ended questions regarding level of satisfaction, enjoyment, importance of program continuity, and continued attendance.

Figure 2 illustrates that across all time points, the majority of respondents preferred an online program format. Respondents were also highly satisfied with the program, indicating a desire for the program to continue (Figure 3). As NeuroSask is a voluntary program, who participates at any given time varies from session to session. At the two-year time point 23% of respondents reported attending “all the time,” 12% “about half the time,” 10% “occasionally,” and 55% “whenever they could.” In the two-year survey, we asked survey participants to identify how long they had been participating with NeuroSask: 39% reported attending “since the beginning,” 26% “for 18 months,” 16% “for 12 months,” 9% “for 6 months,” and 10% “for 3 months.”

On survey 2 and 3, participants were asked to “check all” from a list of potential benefits which included: “Mental wellbeing”, which was reported by 86 and 82% of respondents on survey 2 and 3 respectively; “physical wellbeing” by 97 and 96%; “overall wellbeing” was only asked on survey 3 and selected by 80% of respondents; “sense of support” by 78 and 86% on survey 2 and 3 respectively; “improved symptom management” selected by 58 and 70%; “increased awareness of existing resources” by 65 and 68%, and “other, please specify” was selected by 78 and 86%. The “other” option provided open text data that was included in the qualitative analysis.

### 3.2 Qualitative results

**Figure 4:**
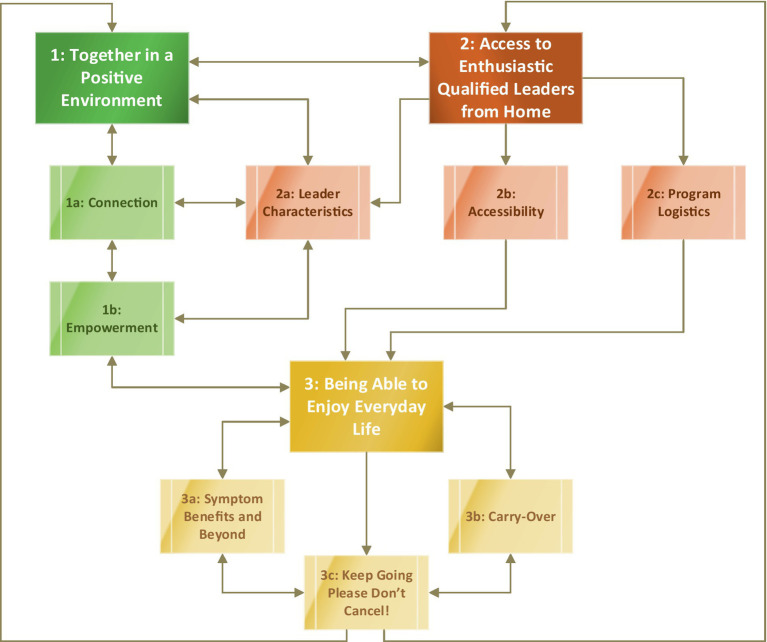
Relationships between themes and subthemes yielded from qualitative analysis across all three time-points. The bidirectional arrows represent a two-way relationship, and the unidirectional arrows represent a one-way relationship. This figure represents interacting relationships between themes and subthemes. Green (theme 1 and corresponding subthemes). Amber (theme 2 and corresponding subthemes). Gold (theme 3 and subthemes).

Reflexive thematic analysis yielded three main themes with eight corresponding subthemes highlighting the prevailing perspectives of the NeuroSask participants: theme 1 “together in a positive and encouraging environment” (subthemes 1a: connection, 1b: empowerment); theme 2 “access to enthusiastic, qualified leaders, from home” (subthemes 2a: leader characteristics, 2b: accessibility, and 2c program logistics); theme 3 “seeing my progress and being able to enjoy everyday life” (subthemes 3a: symptom benefits and beyond, 3b: carry-over, and 3c: keep going, please do not cancel). Table 2 displays select quotations representative of each theme and subtheme. Relationships between the main themes and subthemes are displayed in Figure 4.

**Table 2:** Selected for each major theme and subtheme from the reflexive analysis process.

**Together in a positive environment**	“it brings people together in a positive and encouraging environment” *P212, S3, female, age 40–59, living with MS*
Connection	“I feel I fit in, rather than being the odd person out, the one with the disability. I like that I can get a good amount of exercise safely. I like the mood and the encouraging tone. I like being part of a such a big group!” *P63, S1, Female, age 60+, living with MS* “The class has also provided a sense of social connection during Covid self-isolation, but also from self-isolation and loneliness that happens with MS often anyway.” *P89, S2, female, age 40–59, living with MS* “A sense of belonging. I’ve tried other classes and these by far are the only sessions I look forward to…and I do not feel disabled.” *P127, S1, female, age 20–39, living with a SCI*
Empowerment	“Yes! Positively! NeuroSask makes me feel seen, and like I matter, as if I am worthwhile. So many exercise programmes are only for “able bodied” people and the assumption is often that those defined as having disabilities cannot exercise, would not want to exercise, and/or are not safe to exercise. Most research on physical activity specifically excludes those with walking (and other) mobility limitations/disabilities. NeuroSask is “for” people like me! Sense of personal power rather than giving in. I look forward to all the stretching and I always leave feeling accomplished. I try to do exercises on my own, but I do much more with neurosask, as it is physically and mentally uplifting. The chat after is helpful too, especially to find out how others are challenged and how they cope.” *P418, S3, age 20–39, living with MS* “Something to look forward to. A sense of belonging. I am now able to walk short distances with out a cane, my gait is improving. People have commented on how much I have improved since participating in NeuroSask.” *P166, S3, age 60+, living with PD*
**Access to enthusiastic qualified leaders from home**	“NeuroSask is upbeat, a little quirky, very respectful and full of qualified leadership” *P188, S3, female, age 60+, living with MSA*
Leader characteristics	“I feel a sense of empathy, understanding and encouragement from [the leaders]. The sessions are one of the highlights of my week.” *P23, S2, female, age 40–59, living with MS* “Brilliant! I enjoy the committed time for class. It gives me something to look forward to. I enjoy those who facilitate it. They keep it so enjoyable and informative.” *P211, S3, female, age 60+, living with MS* “The instructors are amazing and their expertise in MS and neurological disorders are second to none. They know how to work with us for maximum benefit and I feel cared about. I love their smiles and the energy they give us.” *P45, S3, female, age 20–39, living with MS*
Accessibility	“I have medical difficulty in joining live exercise programs. The Zoom class is “live” but I can join without leaving my house which is very important to me.” *P180, S3, male, age 60+, living with PPS* “Access right in my home for free. As a person with limited mobility, it keeps me moving. I love that great feeling I get with the exercise.” *P286, S2, female, age 40–59, living with a brain injury* “I love that I can do the exercises “live” with so many others. Thank you for all that you do to include us. It helped me feel more connected! Less alone! A place where I feel like I belong” *P164, S2, female, age 60+, living with a SCI*
Program logistics	“Motivates me to move, even on difficult days. The days and time are regularly scheduled as part of my routine.” *P281, S3, female, age 60+, living with MS* “This program works for me but directions are also given for all ability levels. I love [leader] both when she has her “nerd” hat on or has her “nerd” hat off. And who gets a physio to work with them twice a week. [Other leader] is lovely.” *P47, S3, female, age 60+, living with MS* “I love that the class is live and is adjusted somewhat each time so that we can work our body in different ways. That makes it really engaging and fun every time. Which means that I am encouraged to attend and get this valuable exercise completed. I also love the connect sessions as they provide such a variety of useful information. Overall it is such a wonderful program and I am so grateful that it exists.” *P322, S3, female, age 40–59, living with MS*
**Being able to enjoy everyday life**	“After participating I feel motivated to continue and my day is just better, that mood and energy carry over into what I do afterwards” *P122, S3, female, age 40–59, living with MS*
Symptom benefits and beyond	“How I feel when class is finished. Noticeable improvement in for strength. I love all of it, I cannot seem to come up with anything else. The whole thing is magnificent. Knowing I am helping myself, I especially like that I have a committed time to motivate me. I feel a big difference in my posture and overall strength!” *P311, S3, female, age 60+, living with MS* “it maintained my range of motion and function during the worst relapses I have had. Now I am in remission but NeuroSask is the reason I was able to jump back into regular exercise without hurting myself. The foundation it provided for me is the only reason I am as high functioning as I am now. Basically this program is amazing. I recommend and tell everyone about it.” *P36, S3, female, age 20–39, living with MS*
Carry-over	“It makes life better. Thoroughly enjoy the exercises, even though a lot of the time I do not feel like doing them. I enjoy the connect sessions, there is always something to learn that helps me.” *P293, S3, female, age 60+, living with PD* “I am in a better mood and have a sense of control.” *P26, S1, female, age 40–59, living with cerebellar ataxia* “It has improved my balance and flexibility which allows me to continue to tackle everyday tasks and more importantly tackle new goals like walking up or down stairs.” *P208, S3, female, age 60+, living with MS* “Only program available to take part in your home it helps to address the symptoms I deal with… Stepping in and out of the bathtub every day.” *P150, S3, male, age 60+, living with MS*
“Keep going, please do not cancel”	“Keep on offering this program. It has changed my life. I have recommended this program to others.” *P93, S3, female, age 60+, living with MS* “Thank you so much for what you do! Please do not cancel!” *P91, S3, female, age 40–59, living with MS* “I can say without hesitation that NeuroSask: Active and Connected has given me the desire and strength to take responsibility to manage my life despite the obstacles created by MS as well as the ability to do so.” *P332, S3, female, age 60+, living with MS* “Please continue this program. It is so flexible and I can do it anywhere using whatever device I have” *P381, S3, male, age 60+, living with PD*

Age in years; MS, multiple sclerosis; MSA, multiple systems atrophy; P, participants; PD, Parkinson’s disease; PPS, post-polio syndrome; S, survey time point (1, 2, or 3); SCI, spinal cord injury.

#### 3.2.1 Theme 1: “together in an encouraging environment”

The core theme “together in an encouraging environment” captures the participant reported sense of community and support provided through the NeuroSask program. This theme also encompasses the psychosocial benefits of interpersonal connection, motivation, and empowerment.

##### 3.2.1.1 Subtheme (1a) connection

Central to theme 1 were responses emphasizing the “*connection*” the NeuroSask program provided, majority of respondents directly used the word *“connection.”* Multiple layers of connection were reported: connection to an *“ongoing supportive networ*k” with others through shared experience, connection in the form of *“camaraderie”* and *“socialization,”* and connection through *“empathy,” “encouragement,”* and a *“sense of belonging”*.

Respondents reported that the NeuroSask program provides an opportunity to connect with others living with a neurological condition and facing similar daily challenges. Several respondents reported that a major benefit of NeuroSask is *“knowing you are not alone.”* One respondent noted: “*(it) really helps when you realize others often have the same symptoms as you” (Participant ‘P’128, Survey ‘S’2, female, age 40–59 years, living with a brain injury)* reflecting on the connection to others with a shared experience. Another noted NeuroSask provided a *“supportive network where they fit in rather than being an outlier” (P100, S3, female, age 40–59, stroke survivor),* highlighting how the program provides them with *“a like-group.”* A respondent spoke to the atmosphere of the program: *“there are people who genuinely care and want to help improve our quality of life” (P383, S3, female, age 60+, living with MS).*

Respondents also reported the NeuroSask community provided them with an opportunity for socialization and friendship that they otherwise would not have access to. NeuroSask provided a new social network to participants: “*allowing real communication and social interaction.”*

The “*emotional support”* of the *“positive”* and *“encouraging tone”* of NeuroSask also contributed to respondents’ sense of connection. Respondents felt NeuroSask created a *“sense of community which may be missing from one’s life.”* One respondent noted: *“you are part of a family, the NeuroSask team is very supportive and encouraging” (P53, S3, female, age 40–59, living with MS).* Respondents also valued connecting to others with lived experience through *“socialization from being part of a like group,” “with other people that have challenges on a regular basis like mine.”* The many layers of connection echoed a *“sense of belonging”* which NeuroSask provided for respondents, through connection with program participants and program leaders.

##### 3.2.1.2 Subtheme (1b) empowerment

The majority of respondents reported a sense of *“empowerment”* and/or *“purpose”* provided through program participation. One respondent noted NeuroSask created *“a sense of personal power rather than giving in” (P303, S3, female, age 60+, living with MS)*, highlighting an inspired self-confidence. Another respondent noted *“(NeuroSask) gives me a reason to get up in the morning. It is something to look forward to” (P331, S2, male, age 40–59, living with MS),* speaking to the motivation and inspiration of a regular program. A respondent felt the program provided a “*supportive and fun atmosphere that makes (them) want to rise to the occasion” (P280, S3, female, age 40–59, living with MS).* Attending weekly sessions gave many respondents a “*sense of accomplishment,”* increasing their “*confidence*” and providing “*encouragement to keep coming back and improving.”* Some respondents noted a newfound “*sense of control*,” which in turn lead to a *“much-improved positive outlook”* on their “*situations, abilities*” and their future.

#### 3.2.2 Theme 2: access to “enthusiastic, qualified, leaders from home”

This theme represents the components of the program perceived to facilitate delivery and enhanced participation. Positive and compassionate leaders knowledgeable and experienced in working with individuals living with neurological conditions, combined with an accessible virtual platform that consistently offers regularly scheduled (e.g., on-going) classes were valued by participants.

##### 3.2.2.1 Subtheme (2a) leaders

More than half of the respondents reported on perspectives concerning the program leadership. One respondent felt an *“overwhelming reassurance that participants are with experts who are empathetic, helpful, and fun” (P169, S3, female, age 60+, living with MS).* Respondents reported the leaders created an *“energizing and light atmosphere,”* they were “*remarkably welcoming, their expertise, their commitment and sense of fun are great.”* The attributes of the leaders were described as *“quirky”* and *“qualified”* suggesting that it was the combination of personality attributes and professional skills in the leaders that was perceived as important. Another respondent noted the emotional support provided by the program’s leaders: “*I feel cared about. I love the empathy and the smiles they give us. Sometimes I just join to listen even if I’m not exercising”* (*P22, S3, female, age 40–59, living with MS*).

Respondents noted that *“qualified leadership”* provided a *“resource”* for *“relevant,” “evidence-based medical knowledge.”* More than a third of respondents touched on the insight the program provides with regards to their neurological conditions, as one noted *“(the leaders) taught me so much about living with multiple sclerosis” (P178, S3, female, age 40–59, living with MS)*. Respondents described benefit in having experts in neurorehabilitation lead the active sessions, as well as explain the reasoning behind the therapy in the context of symptoms and challenges experienced by people living with neurological conditions. A respondent noted that the *“leaders discussed important topics, were easy to understand, approachable and willing to answer any questions” (P27, S2, female, age 60+, living with cerebellar ataxia).* When asked what was unique about the program, one respondent said: *“The insight of professionals and medical knowledge given. Where else can I get that top shelf support showing what my disease is and how to manage in a positive manner” (P18, S3, female, age 40–59, living with MS).*

##### 3.2.2.2 Subtheme (2b) accessibility

The subtheme accessibility details the NeuroSask Zoom videoconference format as being easy to access. Approximately a third of respondents mentioned the importance of a *“free live-online session,”* as it provided a *“lifeline support from the comfort of their own home.”* Of the respondents that discussed accessibility, about half noted they faced physical barriers (transportation, rural community or mobility challenges) and therefore *“the exercise and learning available through NeuroSask would be very difficult to access in person.”* The ability to connect to a *“real-time interactive community”* was important to respondents. However, some requested for the connect sessions to be recorded *“record sessions so they can be viewed later or multiple times.”* Respondents consider NeuroSask an accessible *“vital connection”* to information they described as missing or unable to get elsewhere.

##### 3.2.2.3 Subtheme (2c) logistics

There were “logistical” considerations identified as desirable components of the program. Specifically, many respondents found that the *“length of time,” “weekly routine,”* and *“email reminders”* were useful and contributed to their continued attendance. Respondents *“loved the length of time because it was manageable.”* It was noted that having a consistent scheduled time (every Tuesday and Thursday at 2 pm Saskatchewan time year-round) increased participant accountability and commitment. One respondent replied: *“(NeuroSask) motivates me to move, even on difficult days. The days and time are regularly scheduled as part of my routine” (P37, S3, female, age 60+, living with a brain injury).* However, some respondents had difficulty with the afternoon timing of the class due to afternoon fatigue, or their respective local time zones being later, noting *“Afternoon timing does not work for me”*; another responded: *“Could it be an hour or two earlier to help those who attend from the east end of Canada.”* There were also respondents that requested an increase in frequency to *“if 3 times a week is ever possible, that would be awesome: Monday, Wednesday, Friday.”*

Respondents spoke of the importance of having the program lead from a *“seated position,”* as this was inclusive for all. There was frequent mention of the beneficial *“adaptations”* for the various neurological impairments. Respondents perceived modification of the exercises for different abilities as helpful and inclusive. However, two respondents gave feedback that they would prefer a standing position for the class. Many respondents noted they felt that the program was tailored specifically to people with their neurological condition, highlighting the universality of the programing. One respondent is quoted as saying *“It’s designed for people with issues like mine-peripheral polyneuropathy” (P320, S3, female, age 60+, living with sensory peripheral polyneuropathy)*. Another reported “*This program works for my ability and directions are always given for alternate abilities. Not alone in this” (P36, S2, female, age 40–59, living with MS).*

#### 3.2.3 Theme 3: “seeing my progress and being able to enjoy everyday life”

This theme entails the perceived benefits participants of NeuroSask program described, including improvement in symptoms, physical and functional abilities, and quality of life. Respondent’s advocacy for continuation of the program and/or need for such services also speaks to the perceived benefits.

##### 3.2.3.1 Subtheme (3a) symptoms benefits and beyond

The symptom benefits and beyond subtheme encompasses the connections respondents made between program participation and physical experiences in everyday life, including changing disease symptoms. Approximately a third of all responses described *“physical improvements.”* Respondents noted improved balance, flexibility, mobility, increased peripheral muscle strength, increased core strength, increased body awareness and improved range of motion. One response follows*: “(I) found myself more flexible, coordination improved, balance improved” (P216, S3, female, age 40–59, living with MS)*. In addition to the self-reported accounts of improved function, responses also noted pain relief (general and neuropathic), improved sleep, weight loss, spasticity management, and slowing of disease progression. For example, one respondent shared: *“I started the sessions in January while in a mild relapse. And now in June, I feel stronger than I have in years”* (*P33, S3, female, age 40–59, living with MS*). Another noted “I am more limber and stronger which results in my being more confident and more mobile” *(P19, S2, female, age 40–59, living with MS),* and another reported *“it has lessened my stiffness and pain and I can now manage to do many daily living tasks” (P9, S3, female, age 40–59, living with a spinal cord injury).*

##### 3.2.3.2 Subtheme (3b) carry-over

The subtheme carry-over describes the impact of NeuroSask was reported to have on daily activities and function. Several respondents noted that NeuroSask *“has increased their level of independence,” “increased their activity”* outside of the program, and *“improved their quality of life.”* Functional improvement was reported and emphasized as highly important to respondents, including the ability to ambulate, independence with activities of daily living and instrumental activities of daily living. Respondents noted: *“Independence, the ability to have a home, live alone, and maintain a yard” (P230, S3, female, age 40–59, living with MS)*. This concept is also illustrated by the quote *“I have recently lost some mobility due to MS and, because of participating in NeuroSask I have been strong and stable enough to travel a bit, to garden and get around as best as I can” (P268, S3, female, age 60+, living with MS)*. Another respondent spoke to carry-over: *“NeuroSask has helped to improve my individual daily exercise routines, I’m walking a lot more. It motivated me to volunteer in my community. My strength and endurance have improved” (P308, S3, female, age 60+, living with a spinal cord injury).*

##### 3.2.3.3 Subtheme (3c) “keep going please do not cancel”

Approximately one third of the respondents provided comments directly advocating for the program to continue and/or specifically requesting continuation of the program, noting to *“keep it going, please do not cancel!.”* One respondent noted to *“Please, please keep it going!!!!! The good (NeuroSask) does is immeasurable!” (P189, S3, female, age 60+, living with MS).* Another respondent noted *“this program is a true gem for those with disabilities.”* Respondents also discussed the cumulative benefits, *“I am hooked and do not want to lose the momentum NeuroSask has given me”* (*P343, S3, female, age 40–59, living with MS*). Respondents spoke to the significance of NeuroSask: *“Having an exercise program that is realistic and reliable has had a significant impact in my life. I would be very sad without it” (P19, S3, female, age 40–59, living with MS).*

## 4 Discussion

Respondents participating in the NeuroSask program evaluation reported the virtual platform was accessible and the program provided perceived physical benefits, social connection and psychosocial wellbeing. The results from this program evaluation provide only limited information from self-selected individuals. No comparator groups and no objective baseline or follow up data concerning participants utilization of other services, or their physical or psychosocial function are available. However, the perspectives of those who provided feedback may be informative for future program planning. The NeuroSask program was conceptualized during the COVID-19 pandemic, when people with neurological conditions in particular experienced isolation and decreased access to services (20, 21). Based on the survey response data, participants found that a virtual program such as NeuroSask, may create a sense of community and connection by providing an accessible platform for socialization, emotional support and the sharing of information. Freeman et al. suggest that among people with MS, physical restrictions may initially contribute to social isolation, however social experiences and emotional responses may perpetuate isolation (22). Social isolation in people with chronic conditions is associated with poorer health outcomes including cardiovascular comorbidity and related hospitalizations, early mortality, cognitive impairment, and depression (23). In contrast, social connection is associated with positive outcomes in patients with chronic disease (24–27).

Our program evaluation suggests that NeuroSask may provide a desired social network for some people with neurological conditions. Respondents spoke to the camaraderie provided by the NeuroSask cohort, noting the value of a shared experience among people living with similar conditions, symptoms, and/or challenges. Despite the heterogeneity of our participants, respondents felt as though the program was tailored to them individually. Respondents also highlighted the impact that NeuroSask has had on motivation, sense of personal power, and accomplishment. This may suggest benefits extend beyond symptom management to improving daily life experience, and perhaps further informs our understanding of isolation in relation to the psychosocial aspects of chronic neurological disease (20, 21).

Virtual chronic disease management programs can provide participants with similar physical benefits compared to in-person programs, in a safe, and effective manner (28–32). Current literature also supports the growing use of videoconferencing for rehabilitation in neurological conditions to improve physical activity and functional mobility (33–38). Brown et al. conducted a systemic review and meta-analysis of 32 virtual exercise programs delivered in-real time, by a health care professional in an individual or group context. The review demonstrated that videoconferencing is a feasible modality of exercise delivery improving markers of exercise capacity as well as quality of life for people with a chronic disease (33). Findings also suggest that videoconferencing is a suitable delivery platform for exercise training in a wide variety of diseases, and resulted in comparable improvements when compared to in-person interventions (33). Our findings align with this growing body of research on the beneficial use of videoconferencing for people with a neurological condition. Our findings also align with current literature in people with multiple sclerosis that highlights self-esteem, self-efficacy, resilience, and social support contribute to an improved quality of life (39). Negative predictors for quality of life in this population include older age at diagnosis, increased fatigue, lack of disease specific information, lack of social support, fun, or entertainment (40, 41). In certain cases, our respondents reported on the “fun” nature of NeuroSask as well as increased independence, involvement in other physical activities, becoming more active members of their community, and reporting improvements in their mental health. Virtual programs can provide meaningful support and should be considered a be part of routine care (32, 42–44).

People living with a neurological condition may face unique challenges in terms of mobility and transportation that negatively influence access to in-person services (e.g., exercise programming and rehabilitation services). Programs utilizing videoconferencing can increase access to care (14, 29, 30). Our findings emphasize the importance of accessibility from the participant perspective. Many respondents referred to physical barriers such as transportation, mobility, rural location, or cost as limiting participation in programming. Some respondents even noted they are not able to attend in-person programs or services at all. Research by Chan-Nguyen et al. suggests that in general, rural residents favour virtual care (14). However, approximately 20% of respondents self-identified as living rural and about 80% were from a small or large city (Table 1). This may suggest that people with neurological conditions living in non-rural centers may also prefer virtual options over in person programs. Across all time points, including after the pandemic, survey respondents preferred online delivery of the program (Figure 2). NeuroSask was launched as an educational group-based program, and not for the purpose of individualized health care. The COVID-19 pandemic may have helped to reveal a preexisting accessibility issue for people living with neurological conditions. NeuroSask, at least partially for participating individuals, helped to fill a gap in the continuum of chronic disease management.

### 4.1 Limitations and future research

The survey responses from our NeuroSask program evaluation support that participants found the program beneficial. However, the data are limited to those who completed the surveys and who also self-selected to register or participate in the program at all. The program is not a fit for everyone. Virtual programming may enhance accessibility for people with physical impairments, yet virtual programs can still be inaccessible to people experiencing other access barriers (e.g., reliable internet access, device availability, digital literacy, need for physical support to help set-up device and room to access program etc.) (14, 45, 46). Future research could investigate how to best support people with neurological conditions who desire to participate in this type of virtual programming but face technological or logistical barriers. NeuroSask offers a seated-movement class, which may be less appropriate for people with more severe upper extremity or trunk control impairments. People who are fully ambulatory may benefit from virtual standing and balance programing over a seated program. A limitation of the current analysis is that we did not collect any data related to disease severity, physical or cognitive ability, or other social and ethnic demographic information. More information on who participated and benefited from the program would be useful for tailoring the program further. Knowing the audience well could help develop greater program inclusiveness and reach by addressing further specific needs and barriers (e.g., disease severity of participants and/or other attributes that might contribute to participation and effectiveness such as support at home and comfort with technology). Specifically inviting participants and chronic disease service providers to provide perspectives on how to increase the interest and applicability of the program to a wider audience may be valuable.

The findings presented in this study are based solely on participant self-reporting. Although the analysis of participants’ perspectives provides valuable information, quantitative data on health outcomes and function were not available (e.g., cardiometabolic profiles, hospitalizations, comorbidities, fitness level, symptom severity, range of movement etc.). Future research could evaluate the impact of such a program on other measures to help inform the program benefits, identify responders, and potential dose–response relationships. This data could further enhance programming and advocacy for similar services. Integration of virtual chronic disease management support programs by implementation into routine care and sustainable, publicly funded models should be explored, including cost-effectiveness analyses. Gaining insight from people who refer individuals with neurological conditions to the NeuroSask program (e.g., health care providers from different care contexts, program facilitators, partners and connect guests) would also be informative. Multi-source feedback may help to determine the role of such programs in the continuum of care and allow a better understanding of how such programs compliment other existing community and/or chronic disease management services.

Our results are also subject to survey voluntary response and/or non-response bias. The shared perspectives are only representative of those who voluntarily completed the surveys. We do not have information concerning the behaviours and perspectives of people who did not complete the surveys. However, respondents were representative of registered program users with respect to available demographics. Our findings highlight the benefits of NeuroSask from the participants’ perspectives, and their strong desire for the program to continue. NeuroSask was found to be an important program for people living with a neurological condition.

### 4.2 Conclusion

The Neurosask Active and Connected program is an example of a feasible and accessible platform for supporting chronic disease management (i.e., the combination of real-time physical activity, education, and self-management support) for some individuals with neurological conditions. It was perceived as beneficial for fostering community and connection in a positive environment with perceived benefits extending beyond symptom management to participant reported improvements in function, daily life, and disease experience. Although future research will help inform who the program is “best suited” for (vs. who we are not meeting the needs of), efforts should be made to support the creation, implementation, and ongoing evaluation of similar programs for more people with neurological conditions.

## Data availability statement

The raw data supporting the conclusions of this article will be made available by the authors, upon request to the corresponding author.

## Ethics statement

The studies involving humans were approved by Behavioural Research Ethics Board, University of Saskatchewan. The studies were conducted in accordance with the local legislation and institutional requirements. The participants provided their written informed consent to participate in this study.

## Author contributions

SP: Formal analysis, Writing – original draft. KK: Conceptualization, Formal analysis, Funding acquisition, Supervision, Writing – original draft. CE: Conceptualization, Funding acquisition, Writing – review & editing. ML: Conceptualization, Funding acquisition, Writing – review & editing. GL: Conceptualization, Funding acquisition, Writing – review & editing. IP: Conceptualization, Funding acquisition, Writing – review & editing. AR: Conceptualization, Funding acquisition, Writing – review & editing. SD: Conceptualization, Data curation, Formal analysis, Funding acquisition, Project administration, Resources, Supervision, Writing – original draft.

## Footnotes

^1^ https://www.surveymonkey.com/

## References

[1] Organization TWH. Neurological Disorders. Geneva: Public Health Challenges (2006).

[2] Gaskin J Gomes J Darshan S Krewski D. Burden of neurological conditions in Canada. Neurotoxicology. (2017) 61:2–10. doi: 10.1016/j.neuro.2016.05.001, PMID: 27153747

[3] Pringsheim T Fiest K Jette N. The international incidence and prevalence of neurologic conditions: how common are they? Neurology. (2014) 83:1661–4. doi: 10.1212/WNL.0000000000000929 25349272 PMC4223083

[4] Audulv Å Hutchinson S Warner G Kephart G Versnel J Packer TL. Managing everyday life: self-management strategies people use to live well with neurological conditions. Patient Educ Couns. (2021) 104:413–21. doi: 10.1016/j.pec.2020.07.025, PMID: 32819756

[5] Bray G Strachan D Tomlinson M Bienek A Pelletier C. Mapping connections: An understanding of neurological conditions in Canada. Ottawa: Public Health Agency of Canada (2014).

[6] Winberg C Kylberg M Pettersson C Harnett T Hedvall PO Mattsson T. Feeling controlled or being in control? Apps for self-management among older people with neurological disability. Disabil Rehabil Assist Technol. (2021) 16:603–8. doi: 10.1080/17483107.2019.1685017, PMID: 31711351

[7] Rae-Grant A Turner AP Sloan A Miller D Hunziker J Haselkorn JK. Self-management in neurological disorders: systematic review of the literature and potential interventions in multiple sclerosis care. J Rehabil Res Dev. (2011) 48:1087–100. doi: 10.1682/JRRD.2010.08.0159, PMID: 22234713

[8] Kessler D Liddy C. Self-management support programs for persons with Parkinson’s disease: an integrative review. Patient Educ Couns. (2017) 100:1787–95. doi: 10.1016/j.pec.2017.04.011, PMID: 28465112

[9] Jaglal SB Guilcher SJ Bereket T Kwan M Munce S Conklin J. Development of a chronic care model for neurological conditions (CCM-NC). BMC Health Serv Res. (2014) 14:1–2. doi: 10.1186/1472-6963-14-409 25236443 PMC4262116

[10] Davies F Wood F Bullock A Wallace C Edwards A. Shifting mindsets: a realist synthesis of evidence from self-management support training. Med Educ. (2018) 52:274–87. doi: 10.1111/medu.13492, PMID: 29314172

[11] Davies F Wood F Bullock A Wallace C Edwards A. Training in health coaching skills for health professionals who work with people with progressive neurological conditions: a realist evaluation. Health Expect. (2020) 23:919–33. doi: 10.1111/hex.13071, PMID: 32468639 PMC7495084

[12] Clare L Teale JC Toms G Kudlicka A Evans I Abrahams S. Cognitive rehabilitation, self-management, psychotherapeutic and caregiver support interventions in progressive neurodegenerative conditions: a scoping review. NeuroRehabilitation. (2018) 43:443–71. doi: 10.3233/NRE-172353, PMID: 30412509

[13] Brichetto G Tacchino A Leocani L Kos D. Impact of Covid-19 emergency on rehabilitation services for multiple sclerosis: an international RIMS survey. Mult Scler Relat Disord. (2022) 67:104179. doi: 10.1016/j.msard.2022.104179 36130457 PMC9474392

[14] Chan-Nguyen S Ritsma B Nguyen L Srivastava S Shukla G Appireddy R. Virtual care access and health equity during the COVID-19 pandemic, a qualitative study of patients with chronic diseases from Canada. Digital Health. (2022) 8:205520762210744. doi: 10.1177/20552076221074486 PMC8808134 35116172

[15] Boger E Ellis J Latter S Foster C Kennedy A Jones F. Self-management and self-management support outcomes: a systematic review and mixed research synthesis of stakeholder views. PLoS One. (2015) 10:e0130990. doi: 10.1371/journal.pone.0130990, PMID: 26162086 PMC4498685

[16] Braun V Clarke V. Using thematic analysis in psychology. Qual Res Psychol. (2006) 3:77–101. doi: 10.1191/1478088706qp063oa

[17] Braun V Clarke V. One size fits all? What counts as quality practice in (reflexive) thematic analysis? Qual Res Psychol. (2021) 18:328–52. doi: 10.1080/14780887.2020.1769238

[18] Nott M Wiseman L Seymour T Pike S Cuming T Wall G. Stroke self-management and the role of self-efficacy. Disabil Rehabil. (2021) 43:1410–9. doi: 10.1080/09638288.2019.1666431 31560230

[19] Block L Peck M Gilmore-Bykovskyi A. Design and preliminary evaluation of a community-based brain health promotion and wellness program. Innov Aging. (2020) 4:224–5. doi: 10.1093/geroni/igaa057.724

[20] Sepúlveda-Loyola W Rodríguez-Sánchez I Pérez-Rodríguez P Ganz F Torralba R Oliveira DV. Impact of social isolation due to COVID-19 on health in older people: mental and physical effects and recommendations. J Nutr Health Aging. (2020) 24:938–47. doi: 10.1007/s12603-020-1500-7, PMID: 33155618 PMC7597423

[21] Appireddy R Jalini S Shukla G Lomax LB. Tackling the burden of neurological diseases in Canada with virtual care during the COVID-19 pandemic and beyond. Can J Neurol Sci. (2020) 47:594–7. doi: 10.1017/cjn.2020.92, PMID: 32394872 PMC7270482

[22] Freeman J Gorst T Gunn H Robens S. “A non-person to the rest of the world”: experiences of social isolation amongst severely impaired people with multiple sclerosis. Disabil Rehabil. (2020) 42:2295–303. doi: 10.1080/09638288.2018.1557267, PMID: 30657717

[23] Morina N Kip A Hoppen TH Priebe S Meyer T. Potential impact of physical distancing on physical and mental health: a rapid narrative umbrella review of meta-analyses on the link between social connection and health. BMJ Open. (2021) 11:e042335. doi: 10.1136/bmjopen-2020-042335, PMID: 33737424 PMC7978290

[24] Gu J Yang C Zhang K Zhang Q. Mediating role of psychological capital in the relationship between social support and treatment burden among older patients with chronic obstructive pulmonary disease. Geriatr Nurs. (2021) 42:1172–7. doi: 10.1016/j.gerinurse.2021.07.006, PMID: 34419869

[25] Tickle-Degnen L Saint-Hilaire M Thomas CA Habermann B Martinez LS Terrin N. Emergence and evolution of social self-management of Parkinson’s disease: study protocol for a 3-year prospective cohort study. BMC Neurol. (2014) 14:1–2. doi: 10.1186/1471-2377-14-95 24885181 PMC4016672

[26] Buchman AS Boyle PA Wilson RS Fleischman DA Leurgans S Bennett DA. Association between late-life social activity and motor decline in older adults. Arch Intern Med. (2009) 169:1139–46. doi: 10.1001/archinternmed.2009.135, PMID: 19546415 PMC2775502

[27] Xu X Mishra GD Holt-Lunstad J Jones M. Social relationship satisfaction and accumulation of chronic conditions and multimorbidity: a national cohort of Australian women. Gen Psych. (2023) 36:e100925. doi: 10.1136/gpsych-2022-100925 PMC9950967 36844964

[28] Bianchini E Onelli C Morabito C Alborghetti M Rinaldi D Anibaldi P. Feasibility, safety and effectiveness of telerehabilitation in mild-to-moderate Parkinson's disease. Front Neurol. (2022) 13:1100. doi: 10.3389/fneur.2022.909197 PMC9245570 35785358

[29] Knox KB Nickel D Donkers SJ Paul L. Physiotherapist and participant perspectives from a randomized-controlled trial of physiotherapist-supported online vs. paper-based exercise programs for people with moderate to severe multiple sclerosis. Disabil Rehabil. (2022) 45:1147–53. doi: 10.1080/09638288.2022.2055159 35341443

[30] Dennett R Gunn H Freeman JA. Effectiveness of and user experience with web-based interventions in increasing physical activity levels in people with multiple sclerosis: a systematic review. Phys Ther. (2018) 98:679–90. doi: 10.1093/ptj/pzy060, PMID: 29771367

[31] Dlugonski D Motl RW Mohr DC Sandroff BM. Internet-delivered behavioral intervention to increase physical activity in persons with multiple sclerosis: sustainability and secondary outcomes. Psychol Health Med. (2012) 17:636–51. doi: 10.1080/13548506.2011.652640, PMID: 22313192

[32] Dennett R Coulter E Paul L Freeman J. A qualitative exploration of the participants’ experience of a web-based physiotherapy program for people with multiple sclerosis: does it impact on the ability to increase and sustain engagement in physical activity? Disabil Rehabil. (2020) 42:3007–14. doi: 10.1080/09638288.2019.1582717 30907159

[33] Brown RC Coombes JS Rodriguez KJ Hickman IJ Keating SE. Effectiveness of exercise via telehealth for chronic disease: a systematic review and meta-analysis of exercise interventions delivered via videoconferencing. Br J Sports Med. (2022) 56:1042–52. doi: 10.1136/bjsports-2021-105118, PMID: 35715175

[34] Langer A Gassner L Flotz A Hasenauer S Gruber J Wizany L. How COVID-19 will boost remote exercise-based treatment in Parkinson’s disease: a narrative review. NPJ Parkinsons Dis. (2021) 7:1–9. doi: 10.1038/s41531-021-00160-3 33686074 PMC7940641

[35] Isernia S Di Tella S Pagliari C Jonsdottir J Castiglioni C Gindri P. Effects of an innovative telerehabilitation intervention for people with Parkinson's disease on quality of life, motor, and non-motor abilities. Front Neurol. (2020) 11:846. doi: 10.3389/fneur.2020.00846, PMID: 32903506 PMC7438538

[36] Caniça V Bouça-Machado R Ferreira JJ CNS Physiotherapy Study Group Guerreiro D Nunes R. Feasibility and safety of telerehabilitation for physiotherapy interventions in movement disorders patients. Mov Disord Clin Pract. (2021) 8:1144–7. doi: 10.1002/mdc3.13271 34631955 PMC8485610

[37] Vellata C Belli S Balsamo F Giordano A Colombo R Maggioni G. Effectiveness of Telerehabilitation on motor impairments, non-motor symptoms and compliance in patients with Parkinson's disease: a systematic review. Front Neurol. (2021) 12:627999. doi: 10.3389/fneur.2021.627999 34512495 PMC8427282

[38] Cilia R Mancini F Bloem BR Eleopra R. Telemedicine for parkinsonism: a two-step model based on the COVID-19 experience in Milan, Italy. Parkinsonism Relat Disord. (2020) 75:130–2. doi: 10.1016/j.parkreldis.2020.05.038, PMID: 32723588 PMC7286232

[39] Gil-González I Martín-Rodríguez A Conrad R Pérez-San-Gregorio MÁ. Quality of life in adults with multiple sclerosis: a systematic review. BMJ Open. (2020) 10:e041249. doi: 10.1136/bmjopen-2020-041249, PMID: 33257490 PMC7705559

[40] Hosseini Z Homayuni A Etemadifar M. Barriers to quality of life in patients with multiple sclerosis: a qualitative study. BMC Neurol. (2022) 22:174. doi: 10.1186/s12883-022-02700-7, PMID: 35562707 PMC9102679

[41] O'Mahony J Salter A Ciftci-Kavaklioglu B Fox RJ Cutter GR Marrie RA. Physical and mental health-related quality of life trajectories among people with multiple sclerosis. Neurology. (2022) 99:e1538–48. doi: 10.1212/WNL.0000000000200931, PMID: 35948450 PMC9576302

[42] Ghadiri F Naser Moghadasi A Sahraian MA. Telemedicine as a strategic intervention for cognitive rehabilitation in MS patients during COVID-19. Acta Neurol Belg. (2022) 122:23–9. doi: 10.1007/s13760-022-01875-7 35094365 PMC8801040

[43] Sterrenberg K. A virtual group program to improve quality of life for people living with Parkinson’s: an Australian response to the COVID-19 pandemic. World Fed Occup Ther Bull. (2022) 78:53–8. doi: 10.1080/14473828.2021.1887631

[44] McFarland S Coufopolous A Lycett D. The effect of telehealth versus usual care for home-care patients with long-term conditions: a systematic review, meta-analysis and qualitative synthesis. J Telemed Telecare. (2021) 27:69–87. doi: 10.1177/1357633X19862956, PMID: 31394973

[45] Faheem F Zafar Z Razzak A Kalia JS. Implementing virtual Care in Neurology-Challenges and Pitfalls. J Cent Nerv Syst Dis. (2022) 14:117957352211097. doi: 10.1177/11795735221109745 PMC9252001 35795886

[46] Danilewitz M Ainsworth NJ Bahji A Chan P Rabheru K. Virtual psychiatric care for older adults in the age of COVID-19: challenges and opportunities. Int J Geriatr Psychiatry. (2020) 35:1468–9. doi: 10.1002/gps.5372, PMID: 32602134 PMC7361817

