# Supplementary material for: Participants’ perspectives of “NeuroSask: Active and Connect”—a virtual chronic disease management program for individuals with a neurological condition
Source: Front Neurol. 2024 Jan 24;15:1332859. doi: 10.3389/fneur.2024.1332859 (PMC10847521; doi:10.3389/fneur.2024.1332859)
Supplement: Supplementary file 1 [file Data_Sheet_1.docx]

Appendix 1 – Survey Questions

**Survey #1, 10-weeks post-program launch:**

1. Please rate your overall level of satisfaction with the NeuroSask Program (sliding scale 0-100)
2. Should the NeuroSask: Active and connected program continue? (y/n)
3. (If answered yes to question 2) How important do you think it is for the NeuroSask: Active and Connected program to continue running? (sliding scale 0-100)
4. Do you feel you benefited from attending NeuroSask, if so how/in which ways? (open text)
5. Was there a topic(s) you found particularly important/useful? (open text)
6. Do you have any other suggestions or comments? (open text)
7. Which neurological condition do you have? (multiple choice, a) multiple sclerosis, b) Parkinson’s disease, c) spinal cord injury, d) other, please specify)
8. What is your sex: (multiple choice, a) female, b) male, c) prefer not to answer)
9. What age range (years) do you fall into: (multiple choice, a) less than 20, b)20-39, c) 40-59, d) 60+)
10. Which Province or Territory do you live in? (multiple choice list of Canadian Provinces and Territories)
11. Do you live: (multiple choice, a) rurally, b) small city, c) large city)
12. Outside of COVID-19/the pandemic, do you prefer: (multiple choice, a) online movement class, b) in-person, c) no preference)
13. If NeuroSask were to continue running, would you continue to attend (yes/no)
14. I consent to having my feedback (de-identified/anonymously) shared by the NeuroSask team as part of program evaluation reports and/or future funding applications (yes/no)

**Survey #2, 1-year post-program launch:**

1. Please rate your overall level of satisfaction with the NeuroSask Program (sliding scale 0-100)
2. Should the NeuroSask: Active and connected program continue? (y/n)
3. *(If answered yes to question 2)* How important do you think it is for the NeuroSask: Active and Connected program to continue running? (sliding scale 0-100)
4. Does attending NeuroSask provide any of the following benefits*? (check all that apply: a) mental wellbeing, b) physical wellbeing, c) sense of support, d) symptom management, e) increased awareness of existing resources, f) other, please specify (open text)) **Note these options were informed by the results of survey 1*
5. Was there a topic(s) you found particularly important/useful? (open text)
6. Do you have any other suggestions or comments? (open text)
7. Are you a: (*multiple choice a) person living with a neurological condition, b) care giver, c) health care provider, d) other (please specify). **Note these options were informed by the results of survey 1*
8. Which neurological condition do you have? (*multiple choice: a) multiple sclerosis, b) Parkinson’s disease, c) spinal cord injury, d) stroke, e) brain injury, f) cerebral palsy, g) cerebellar ataxia, h)other (please specify)) **Note these options were informed by the results of survey 1*
9. What is your sex: (multiple choice, a) female, b) male, c) other, d) prefer not to answer)
10. What age range (years) do you fall into: (multiple choice a) less than 20, b)20-39, c) 40-59, d) 60+)
11. Do you live in: (multiple choice, a) Canada, b) other, please specify) – *this question was added based on results of survey 1*
12. *(If answered Canada in question 12)* Which Province or Territory do you live in? (multiple choice list of Canadian Provinces and Territories)
13. Do you live: (multiple choice) a) rurally, b) small city, c) large city
14. Outside of COVID-19/the pandemic, do you prefer: (multiple choice: a) online movement class, b) in-person, c) no preference?
15. If NeuroSask were to continue running, would you continue to attend (yes/no)
16. I consent to having my feedback (de-identified/anonymously) shared by the NeuroSask team as part of program evaluation reports and/or future funding applications (yes/no)

**Survey #3, 2-years post-program launch:**

1. Please rate your overall level of satisfaction with the NeuroSask Program (sliding scale 0-100)
2. Should the NeuroSask: Active and connected program continue? (y/n)
3. *(If answered yes to question 2)* How important do you think it is for the NeuroSask: Active and Connected program to continue running? (sliding scale 0-100)
4. Do you feel you benefited from attending NeuroSask? (yes/no)
5. On average, how often did you attend? (multiple choice, a) whenever I can, b) always, c) occasionally, d) a few times, e) I don’t attend personally, but refer people to the program)
6. Does attending NeuroSask provide any of the following benefits*? (check all that apply: a) mental wellbeing, b) physical wellbeing, c) overall wellbeing d) sense of support, e) symptom management, f) increased awareness of existing resources, g) other, please specify (open text)) **Note these options were informed by the results of survey 1 and 2*
7. Has NeuroSask impacted what you do in your daily life, and if so, how? (open text)
8. What makes NeuroSask special to you/ what do you like most about the program? (open text)
9. Was there a topic(s) you found particularly important/useful? (open text)
10. What influences your motivation to participate in the NeuroSask program/ what keeps you coming back? (open text)
11. Do you have any other suggestions or comments? (open text)
12. Are you a: (*multiple choice a) person living with a neurological condition, b) care giver, c) health care provider, d) other (please specify). **Note these options were informed by the results of survey 1 and 2*
13. Which neurological condition do you have? (*multiple choice: a) multiple sclerosis, b) Parkinson’s disease, c) spinal cord injury, d) stroke, e) brain injury, f) cerebral palsy, g) cerebellar ataxia, h) other, please specify) **Note these options were informed by the results of survey 2*
14. What is your sex: (multiple choice, a) female, b) male, c) other, d) prefer not to answer)
15. What age range (years) do you fall into: (multiple choice a) less than 20, b)20-39, c) 40-59, d) 60+)
16. Do you live in: (multiple choice, a) Canada, b) other, please specify) – *this question was added based on results of survey 1 and kept based on results of survey 2*
17. *(If answered Canada in question 13)* Which Province or Territory do you live in? (multiple choice list of Canadian Provinces and Territories)
18. Do you live: (multiple choice) a) rurally, b) small city, c) large city
19. Outside of COVID-19/the pandemic, do you prefer: (multiple choice: a) online movement class, b) in-person, c) no preference?
20. Roughly how long have you been participating in our NeuroSask program? (multiple choice, a) since the beginning, b) last 3 months, c) last 6 months, d) last 12 months, e) last 18 months)
21. If NeuroSask were to continue running, would you continue to attend (yes/no)
22. I consent to having my feedback (de-identified/anonymously) shared by the NeuroSask team as part of program evaluation reports and/or future funding applications (yes/no)
